# Development of a Sensitive Electrochemical Enzymatic Reaction-Based Cholesterol Biosensor Using Nano-Sized Carbon Interdigitated Electrodes Decorated with Gold Nanoparticles

**DOI:** 10.3390/s17092128

**Published:** 2017-09-15

**Authors:** Deepti Sharma, Jongmin Lee, Junyoung Seo, Heungjoo Shin

**Affiliations:** Department of Mechanical Engineering , Ulsan National Institute of Science and Technology (UNIST), 50 UNIST-gil, Ulsan 44919, Korea; drdeepti2581@unist.ac.kr (D.S.); gmcljw@unist.ac.kr (J.L.); tjwnsdud91@unist.ac.kr (J.S.)

**Keywords:** gold nanoparticles, interdigitated electrodes, electrochemical-enzymatic redox cycling, covalent immobilization, cholesterol sensor

## Abstract

We developed a versatile and highly sensitive biosensor platform. The platform is based on electrochemical-enzymatic redox cycling induced by selective enzyme immobilization on nano-sized carbon interdigitated electrodes (IDEs) decorated with gold nanoparticles (AuNPs). Without resorting to sophisticated nanofabrication technologies, we used batch wafer-level carbon microelectromechanical systems (C-MEMS) processes to fabricate 3D carbon IDEs reproducibly, simply, and cost effectively. In addition, AuNPs were selectively electrodeposited on specific carbon nanoelectrodes; the high surface-to-volume ratio and fast electron transfer ability of AuNPs enhanced the electrochemical signal across these carbon IDEs. Gold nanoparticle characteristics such as size and morphology were reproducibly controlled by modulating the step-potential and time period in the electrodeposition processes. To detect cholesterol selectively using AuNP/carbon IDEs, cholesterol oxidase (ChOx) was selectively immobilized via the electrochemical reduction of the diazonium cation. The sensitivity of the AuNP/carbon IDE-based biosensor was ensured by efficient amplification of the redox mediators, ferricyanide and ferrocyanide, between selectively immobilized enzyme sites and both of the combs of AuNP/carbon IDEs. The presented AuNP/carbon IDE-based cholesterol biosensor exhibited a wide sensing range (0.005–10 mM) and high sensitivity (~993.91 µA mM^−1^ cm^−2^; limit of detection (LOD) ~1.28 µM). In addition, the proposed cholesterol biosensor was found to be highly selective for the cholesterol detection.

## 1. Introduction

Cholesterol is an important biological compound produced in the liver and intestines, where it acts as a biosynthetic precursor of bile acids and vitamin D [1]. It is also an essential component of cell membranes and steroid hormones, and regulates the development of the brain as well as the nervous and immune system in humans [2]. The normal concentration of free cholesterol in human serum is below 5.2 mM, but if its level exceeds 6.2 mM, that could result in a serious health threat, such as coronary disease, arteriosclerosis, myocardial infarction, brain thrombosis, lipid metabolism dysfunction, hypertension, or cerebral thrombosis [3,4]. Its quantification in routine clinical laboratory tests enables the diagnosis and management of various life-threatening diseases [5]. Various analytical techniques have been employed for the determination of blood cholesterol levels, such as gas-liquid chromatography [6,7], high-performance liquid chromatography (HPLC) [8,9], and colorimetric determination [10]. However, these methods have several disadvantages, such as poor specificity and selectivity because of the interfering reactions, time-consuming sample preparation, instability of the color reagents, corrosive nature of the reagent, difficult standardization, and large amount of serum sample [11]. This has led to a great interest in the development of a cost-effective cholesterol biosensor with fast, sensitive, and selective response [12].

Electrochemical cholesterol biosensors based on enzymes have received great attention due to their high sensitivity, selectivity, rapidity, portability, and low cost [13,14]. The key factors focused during the preparation of these biosensors involve the use of active electrode materials with rapid electron transfer and high electrical conductivity, along with efficient and stable enzyme immobilization [15,16]. Most of these amperometric methods are based on monitoring the electrooxidation current of hydrogen peroxide (H_2_O_2_) produced during the catalytic oxidation of cholesterol via cholesterol oxidase (ChOx) [3,17]. ChOx is a flavin adenine dinucleotide (FAD) containing flavoenzyme that catalyzes the oxidation of cholesterol to cholest-4-en-3-one [18]. The oxidation of H_2_O_2_ requires high-applied potential [19] that may lead to lower sensitivity and selectivity due to the oxidation of other interfering species in samples [20]. Therefore, in view of lowering the applied potential and avoiding the influence of reductants such as acetaminophen, ascorbic acid, and uric acids, electron mediators were employed [21,22,23]. Recently, some research studies on electrochemical cholesterol biosensors have focused on the direct electron transfer between ChOx and electrode surfaces at a lower potential to avoid the interference from the co-existing species [12]. However, some problems, including the poor compatibility of the support matrix with the enzyme [24,25], deep embedment of redox active site in the protein, and leaching of the enzyme from the electrode surface, may restrict the analytical efficiency of the developed biosensor [14].

Immobilization of an enzyme onto the electrode surface is a crucial step for the construction of enzyme-based biosensors because substrate diffusion to the active site of the enzyme plays an important role in biosensor performance [11,23]. The commonly used methods for immobilization of ChOx enzymes are entrapment [26,27], physical adsorption [28], and covalent binding using cross-linkers [29]. Physical adsorption is simple and fast, but it suffers from the desorption of proteins during operation or by changes in pH, temperature, and ionic strength. Moreover, to immobilize enzymes via entrapment, the development of a suitable matrix that can hold the enzyme without leaching is highly necessary. On the other hand, the covalent immobilization method was employed to improve uniformity, density, and distribution of the bound protein, as well as reproducibility on the electrode surface [30].

Owing to the unique properties such as good electrical conductivity, large surface to volume ratio, and excellent biocompatibility, nanomaterials can offer new opportunities for the development of biosensors with high analytical performance [31,32,33,34]. It has been reported that ChOx has been immobilized on different platforms, especially on the top of metal nanoparticles (NPs) such as AuNPs [4,35,36,37], AgNPs [38], PdNPs [39], and Pt-Pd hybrid nanocomposites [40]. Gold nanoparticles attracted huge scientific and technological interest due to their ease of synthesis, chemical stability, and quantum size effects, which lead to unique optical, electronic, and catalytic properties. AuNPs enhance the electrode conductivity and facilitate the electron transfer, thus improving the analytical sensitivity and selectivity [41,42,43]. In addition, due to their high surface-to-volume ratio, they increase the electroactive surface area by several orders of magnitude, which results in higher enzyme loading and ultimately leads to high sensitivity within the resulting device [34].

In this study, in view of the advantageous features AuNPs, a sensitive electrochemical biosensor was constructed by using nano-sized carbon interdigitated electrodes (IDEs) decorated with electrochemically deposited AuNPs and employing cholesterol as the model analyte. In the previous study, we explored the nano-sized carbon IDEs as an electrochemical-enzymatic redox cycling-based glucose biosensor system [44]. The carbon IDEs were fabricated using a batch microfabrication technology known as the carbon microelectromechanical system (C-MEMS) process, as reported earlier [45]. Due to the 1:1 aspect ratio and micrometer-sized inter-electrode distance, nano-sized carbon IDEs showed high redox cycling efficiency, and hence high current amplification [45,46]. Despite these advantages, carbon IDEs possesses a major disadvantage that the electrical conductivity of the carbon electrodes lies two to three orders of magnitude below that of most metals [47], thereby exhibiting lower electrochemical performance.

The aim of this study is to develop a highly sensitive biosensor system by enhancing the electroactive surface area, electrochemical activity, and electrical conductivity of carbon IDEs via simple AuNP electrodeposition. One of the most significant advantages of electrochemical deposition is the ability to control the size and distribution of nanoparticles by simply varying potential, time, or solution concentration [48]. Gold nanoparticles can be deposited in a short time period at a high growth rate, and their deposition can deliberately localized at electrode surfaces with minimum material waste. In the present study, the enzyme (ChOx) was covalently and selectively immobilized on a single comb (comb 1) of IDEs using amidation between NH_2_ terminus of enzyme and COOH terminus of 4-carboxymethyl diazonium layer making use of the carbodiimide chemistry [49,50]. Thus, the other comb (comb 2) maintained its electrochemical reactivity during the selective immobilization step; this ensured efficient redox current collection at comb 2. Therefore, both the combs (comb 1 and comb 2) are working electrodes in the presented scheme of biosensor fabrication, and an external Pt wire and Ag/AgCl are used as the counter and reference electrode, respectively. The amperometric detection of cholesterol was performed, using ferricyanide ([Fe(CN)_6_]^3−^) as a redox mediator.

**Scheme 1:**
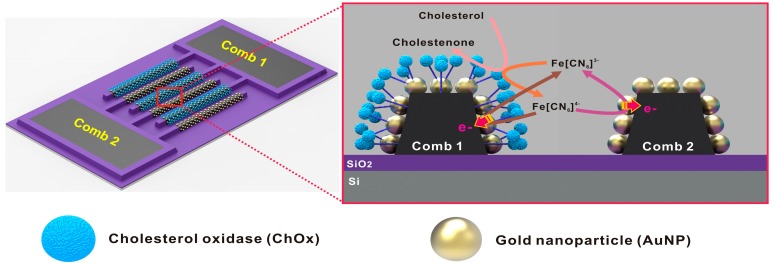
Schematic diagram of the sensing principle based on the redox cycling of [Fe(CN)_6_]^3−^/[Fe(CN)_6_]^4−^ between enzyme and electrode surfaces at nano-sized gold nanoparticle (AuNP)/carbon interdigitated electrodes (IDEs) selectively functionalized with cholesterol oxidase (ChOx); Working electrodes (comb 1: enzyme-functionalized AuNP/carbon comb, comb 2: non-functionalized AuNP/carbon comb).

In this biosensor configuration as shown in Scheme 1, the reaction mechanisms at the enzyme surfaces involve (1) the oxidation of substrate to product, and (2) the reduction of ferricyanide ([Fe(CN)_6_]^3−^) to ferrocyanide ([Fe(CN)_6_]^4−^). The ferrocyanide formed via enzymatic reaction subsequently becomes oxidized at both combs (comb 1 and 2) of the AuNP/carbon IDEs (Scheme 1). The analytical performances of the AuNP-decorated carbon IDEs biosensor were compared with those of recently reported cholesterol biosensors. The advantages of the proposed biosensor system in terms of sensor structure and fabrication include (i) the use of reproducible, easy, and cost effective wafer-level batch nanofabrication technologies (i.e., C-MEMS and electrodeposition), (ii) simple AuNP electrodeposition in short-time, minimizing material waste, and (iii) IDEs with a 1:1 aspect ratio integrated with large surface-to-volume ratio AuNPs, which enable efficient redox cycling. In addition, the AuNP/carbon IDE-based cholesterol biosensor exhibited high sensitivity, wide linear dynamic range, a significantly low detection limit, and good selectivity and reproducibility in cholesterol sensing.

## 2. Experimental

### 2.1. Chemicals and Reagents

An Au thiosulfate-sulfite plating solution (16 g/L) (TG Micro 500), which was used for the deposition of AuNPs, was purchased from T&I CHEM Ltd. (Incheon, Korea). 4-aminophenylacetic acid (4-carboxymethylaniline), potassium ferricyanide (K_3_[Fe(CN)_6_]), potassium ferrocyanide (K_4_[Fe(CN)_6_]), potassium chloride (KCl), sodium nitrite (NaNO_2_), *N*-hydroxysuccinimide (NHS), *N*-(3-dimethylaminopropyl)-*N*′-ethylcarbodiimide hydrochloride (EDC), Triton X-100, cholesterol oxidase derived from *Streptomyces* sp. (≥20 units/mg), cholesterol (Ch), ascorbic acid (AA), uric acid (UA), salicylic acid (SA), acetaminophen (AP), glutathione (GN), glucose (GU), and creatinine (CN) were purchased from Sigma Aldrich Co. (St. Louis, MO, USA). Phosphate-buffered saline (PBS, pH 7.4, 10×) was purchased from Life Technologies (Seoul, Korea). All reagents were of analytical grade and were used without further purification. All solutions were freshly prepared before use. Negative photoresist SU-8 2002, which was used for the fabrication of the nano-sized carbon IDEs, was purchased from Microchem Corp. (Westborough, MA, USA).

### 2.2. Fabrication of Nano-Sized AuNP/Carbon IDEs

Figure S1 illustrates the overall fabrication steps of nano-sized carbon IDEs decorated with AuNPs. In this study, nano-sized carbon IDEs were patterned on a six-inch Si-wafer in a batch manner. First, the wafer was passivated using thermal silicon oxidation (1 µm SiO_2_). After a negative photoresist (SU-8 2002) layer was spin-coated on the SiO_2_/Si wafer, polymer IDEs structures were patterned (width = 1.5 µm, thickness = 3.5 µm, gap = 1 µm) using UV lithography. Using a polymer pyrolysis process at 900 °C under vacuum, the micro-sized polymer IDEs structures were converted into glassy carbon IDEs with an aspect ratio of 1:1 because of the size reduction in pyrolysis by ~90-fold (width = 620 nm, thickness = 650 nm, gap = 1.9 µm). The carbon structures were passivated using multiple insulating layers of 100-nm SiO_2_/100-nm Al_2_O_3_/100-nm SiO_2_, except for a sensing area of 100 µm × 300 µm, which was patterned using a lift-off process that included UV lithography of negative photoresist (NR5-8000, Futurrex, Inc., Franklin, NJ, USA) and subsequent RF sputtering (SRN-120D, SORONA, Pyeongtaek, Korea). To enhance the electrochemical current signal, AuNPs were deposited selectively on the 1:1 aspect ratio nano-sized carbon IDEs by electrodeposition in a diluted (5 mM) Au thiosulfate-sulfite plating solution, using a potentiostat (IviumStat, Ivium Technologies B.V., AJ Eindhoven, Netherlands). The electrodeposition process consisted of two steps: an initial step with a high bias of –0.9 V vs. Ag/AgCl for 20 s, for the nucleation of AuNPs; and a second step with a low bias of –0.7 V vs. Ag/AgCl for 40 s, for the conformal nanoparticle growth. Next, the AuNP-modified carbon IDEs were thoroughly rinsed using deionized (DI) water and dried using N_2_ gas.

### 2.3. Preparation of Enzyme-Functionalized IDEs

**Figure 1:**
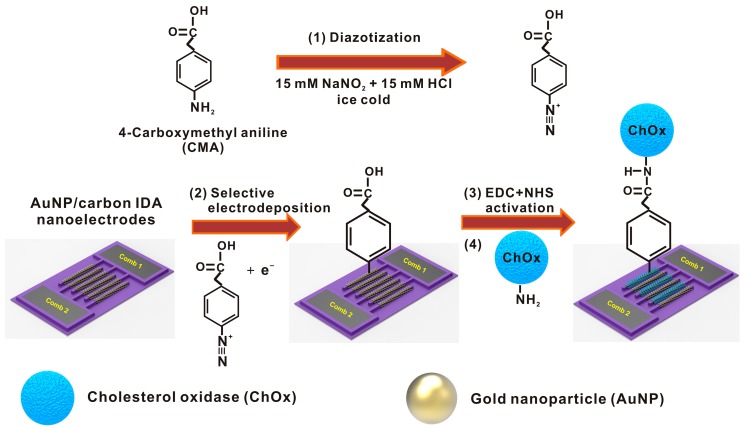
Steps for covalent immobilization of enzyme on the electrode surface (EDC: 1-Ethyl-3-(3-dimethylaminopropyl)-carbodiimide, NHS: *N*-Hydroxysuccinimide, ChOx: Cholesterol oxidase).

The selective immobilization of an enzyme at only one IDE comb was conducted via electrochemically reducing the 4-carboxymethyl diazonium cation. In contrast to previous work [44], presently we used the zero-length crosslinking reagent (EDC-NHS) in order to simplify the scheme of enzyme immobilization. We generated 4-carboxymethyl diazonium salt in situ by treating 20 mM of 4-carboxymethylaniline (CMA) in an aqueous solution of 15 mM HCl and 15 mM NaNO_2_, standing in a beaker of ice for 20 min, under stirring. After this treatment, the –NH_2_ group of the aniline derivative was converted to a –N_2_^+^ group, also known as a diazonium ion (Figure 1, Step 1). The diazonium salt solution was then immediately used for electro-addressing on the one comb of IDEs (Figure 1, Step 2). Two cycles of cyclic voltammetry (CV) were performed from 0.5 to −1.5 V vs. Ag/AgCl, at a scan rate of 200 mV/s, for the selective immobilization of diazonium salt at only one comb of the IDEs, using a multi-potentiostat (CHI 1020; CH Instruments, Inc., Austin, TX, USA). Next, the IDEs were rinsed with DI water, and incubated for 90 min in an activating solution composed of 0.1 M EDC and 0.1 M NHS (Figure 1, Step 3). After the incubation, the IDEs were rinsed with DI water, and dried using N_2_ gas. To immobilize the enzyme (ChOx) with the activated carboxyl (COOH) groups of bound 4-carboxymethyl diazonium moiety, the IDEs were kept in a 50 mM PBS buffer (pH 7.4) solution containing ChOx (4 mg/mL), overnight at 4 °C (Figure 1, Step 4).

### 2.4. Instruments and Measurements 

The scanning electron microscope (SEM) images of bare carbon and AuNP/carbon IDEs were obtained using an S-4800 (Hitachi High Technologies, Tokyo, Japan). The electrochemical reactivity of both combs of the AuNP/carbon IDEs, functionalized with and without ChOx enzymes, were characterized at every functionalization step thereafter, using cycling voltammetry (CV) in the potential range of 0 to 0.8 V vs. Ag/AgCl (reference electrode), in PBS solution (pH 7.4) with 10 mM [Fe(CN)_6_]^4−^, at a scan rate of 50 mV/s. A Pt wire was used as the counter electrode (Figure S2). Cholesterol sensing was performed using chronoamperometry at 0.6 V vs. Ag/AgCl in 10 mM [Fe(CN)_6_]^3−^ (redox mediator) and 50 mM PBS (pH 7.4) solution containing various concentrations of cholesterol. Cholesterol stock solution (0.1 M) was prepared in 0.1 M PBS containing 5% (*w*/*v*) of Triton X-100 and isopropyl alcohol and then kept at 60 °C for 1 h. All electrochemical detections were carried out using a multi-potentiostat (CHI 1020; CH Instruments, Inc., Austin, TX, USA).

## 3. Results and Discussion

### 3.1. Optimization of AuNP Deposition on Carbon IDEs

The AuNPs were electrodeposited on nano-sized bare carbon IDEs using a two-step procedure of nucleation and growth at different potentials in order to ensure the uniform, dense, and conformal growth of AuNPs. The nucleation of AuNPs on nano-sized bare carbon IDEs was optimized at various voltages (−0.6 V to −0.9 V) for 20 s, as shown in Figure S3. The AuNPs at −0.9 V for 20 s (Figure S3D) showed a uniform nucleation of AuNPs all over the carbon IDE surface. The nucleation for more than 20 s resulted in a non-uniform deposition of AuNPs.

Furthermore, the growth potential and time for the AuNPs deposition were also optimized as shown in the Figure S4. We reduced the electrodeposition potential condition to −0.7 V at the growth step to control precisely the AuNP size. We have optimized the growth time while maintaining the nucleation condition (−0.9 V vs. Ag/AgCl for 20 s) and growth potential (−0.7 V vs. Ag/AgCl). As shown in the Figure S4, size, uniformity and density of AuNPs increase with increasing the growth time. However, the deposition time was limited to 40 s to prevent the electrical connection between the two combs of IDEs. In addition, the effect of the growth time on the electrical current signal was evaluated using CV in a solution of 10 mM [Fe(CN)_6_]^4−^ with 0.1 M KCl, as shown in Figure S5. The CV results indicated that as the size of the AuNPs increases, the surface reactivity of the AuNP/carbon IDEs increases correspondingly (Figure S5A). In addition, the current amplification of the [Fe(CN)_6_]^4−^/[Fe(CN)_6_]^3−^ redox species also increased with increasing growth time (Figure S5B). Detailed description about the electrochemical characterization of AuNP/carbon IDEs will be presented in the next section. In conclusion, we selected −0.9 V vs. Ag/AgCl for 20 s for the nucleation condition, and −0.7 V vs. Ag/AgCl for 40 s for the conformal growth of AuNPs after ensuring a maximum redox cycling effect while preventing electrical connection between combs.

### 3.2. Morphological and Electrochemical Characterization of AuNP/Carbon IDEs

**Figure 2:**
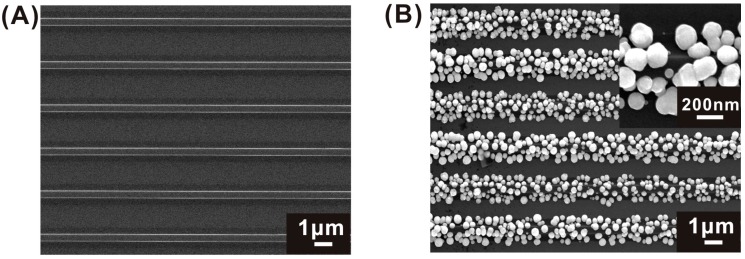
Scanning electron microscope (SEM) images of (**A**) bare carbon IDEs and (**B**) AuNP/carbon IDEs.

AuNPs were electrodeposited on nano-sized bare carbon IDEs using −0.9 V vs. Ag/AgCl for 20 s for nucleation, and −0.7 V vs. Ag/AgCl for 40 s for conformal growth, and their morphologies were characterized using SEM (Figure 2A,B). AuNPs with an average size of 90–150 nm were deposited on the surfaces of the carbon IDEs (Figure 2B). Energy dispersive X-ray (EDX) spectrum analysis confirms that the electrodeposited nanoparticles on the carbon nanoelectrodes are mainly composed of gold, as shown in Figure S6. The AuNP deposition on the sidewalls of carbon nanoelectrodes reduced the inter-electrode distance, which is usually necessary for efficient redox cycling [51].

**Figure 3:**
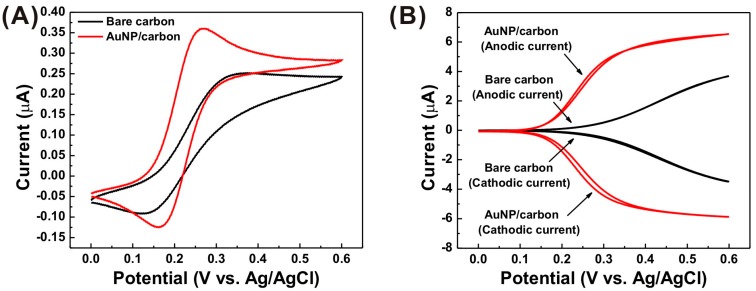
Cyclic voltammograms of the bare carbon IDEs and AuNP/carbon IDEs in 10 mM [Fe(CN)_6_]^4-^ with 0.1 M KCl in (**A**) the single mode (without redox cycling) and (**B**) the dual mode (with redox cycling).

The effect of AuNPs on the electrochemical current signal was characterized using CV in a solution of 10 mM [Fe(CN)_6_]^4−^ with 0.1 M KCl (Figure 3). After the AuNP deposition, the separation between the peak potentials (ΔEp = Epa − Epc; Epa: anodic peak potential; Epc: cathodic peak potential) reduced (from 190 to 85 mV). The peak currents were enhanced in the single mode, where both combs of the IDEs were scanned simultaneously under the same conditions (0 to 0.6 V vs. Ag/AgCl; 50 mV/s), as shown in Figure 3A. This observation indicates that AuNPs enhance the electron transfer rate at the electrode surfaces. However, the saturated current level did not differ significantly after AuNP deposition, because AuNPs consumes the redox species faster at the top surfaces; thus, the effect of the sidewalls of AuNPs and buried carbon surfaces may not be significant. The effect of AuNPs became more noticeable in the dual mode, where a single comb of the IDEs (generator) was scanned from 0 to 0.6 V, while the other comb (collector) was biased at −0.3 V. In the dual mode, the redox species (ferrocyanide in this study) oxidized at the generator and diffused to the neighboring comb or the collector. Next, the oxidized species reduced back to its original state. These redox events are recycled if the inter-electrode gap is small [45]. Significant increases in current amplification (dual mode current/single mode current) occurred at the AuNP-integrated IDEs, relative to bare carbon IDEs (Figure 3B). The diffusion-limited current in the dual mode depends on the electrode gap that was reduced by the size of electroplated AuNPs, and resulted in an increase in the concentration gradient. In addition, owing to the high electrical conductivity of AuNPs, the integration of AuNPs reduced the IR drop effect that occurs when the electrical current flows through IDEs with relatively low electrical conductivity in the dual mode [45]. Thus, the dual mode currents at the AuNP/carbon IDEs reach the diffusion limited saturation currents earlier than bare carbon IDEs.

**Figure 4:**
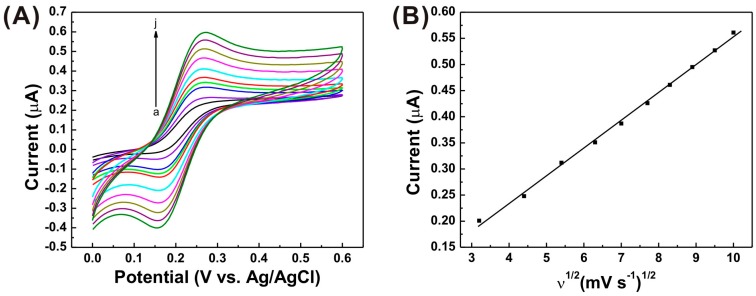
(**A**) Cyclic voltammograms of AuNP/carbon IDEs in 10 mM [Fe(CN)_6_]^4−^ with 0.1 M KCl at various scan rates from curve a to j corresponding to 10, 20, 30, 40, 50, 60, 70, 80, 90, 100 mV/s. (**B**) The relationship between the peak currents and the square roots of the scan rates.

Figure 4A depicts the CVs of the AuNP/carbon IDEs in 10 mM [Fe(CN)_6_]^4−^ with 0.1 M KCl at different scan rates. It was observed that the peak currents are dependent on the scan rate in the range from 10 to 100 mV/s. In addition, the peak current vs. the square root of the scan rate plots (Figure 4B) exhibits a linear relationship, suggesting a quasi-reversible diffusion-controlled process.

### 3.3. Characterization of Selective Enzyme Immobilization

For the selective immobilization of the enzymes (ChOx), one comb (comb 1) of the AuNP/carbon IDEs was modified by the reduction of the aryl diazonium salt. After the diazotization using 15 mM NaNO_2_ and 15 mM HCl in ice-cold solution, the 4-carboxymethyl diazonium salt was reduced specifically on one comb (comb 1) of the AuNP/carbon IDEs using cyclic voltammetry (Figure S7). The first scan in the graph exhibits an irreversible reduction peak at −0.3 V. This feature corresponds to the typical electrochemical reduction wave of the diazonium salt [52], and leads to the elimination of a nitrogen molecule and the production of an aryl radical. This radical attacks the electrode surface and forms a covalent bond between the aryl group and the electrode material [53]. Further, at the second scan, the first irreversible reduction peak has diminished, indicating that the electrode surface was saturated with the grafted 4-carboxymethyl diazonium moieties.

For localizing electrochemical-enzymatic production and the redox cycling of [Fe(CN)_6_]^3−^/[Fe(CN)_6_]^4−^ species in the vicinity of electrode surfaces, ChOx were selectively immobilized on specific comb (comb 1) of the IDEs. Electrochemical characterization of both functionalized and non-functionalized combs (comb 1 and 2) of AuNP/carbon IDEs was performed using cyclic voltammetry in a PBS solution (pH 7.4) containing 10 mM [Fe(CN)_6_]^4−^ after every step of electrode surface modification (Figure S8). As shown in Figure S8A, after modification with the 4-carboxymethyl diazonium salt, the cyclic voltammograms collected from comb 1 exhibited a dramatic decrease in the oxidation current due to the hydrophobic nature of the terminal –COOH groups of the 4-carboxymethyl diazonium salt blocking the diffusion of the [Fe(CN)_6_]^4−^ redox species [54]. The peak currents reduced further after the binding of the EDC and NHS, and the immobilization of the ChOx enzyme at the comb 1 of the AuNP/carbon IDEs. The reduction in peak current may be due to the impedance of several layers of the ChOx enzyme, and their negative charges, at the surface of comb 1. Meanwhile, no significant peak current changes were observed at the non-functionalized comb 2 surface after the 4-carboxymethyl diazonium moiety modification at comb 1, and the EDC and NHS activation steps (Figure S8B). The peak current at the surface of comb 2 slightly reduced only after ChOx enzyme immobilization; this may be due to its non-spontaneous physical binding. This observation confirms that the ChOx enzyme was selectively immobilized on comb 1, while comb 2 retains the electrochemical reactivity, even after the immobilization procedures performed on comb 1. The effect of selective enzyme immobilization on sensing capability will be discussed later.

### 3.4. Optimization of Experimental Conditions for Sensing

The activity of ChOx is affected by the medium pH and applied potential; therefore, the effects of both were examined by measuring chronoamperometric responses of the AuNP/carbon IDE-based biosensor with 0.1 mM cholesterol in 10 mM [Fe(CN)_6_]^3−^/50 mM PBS. As shown in Figure S9A, for the AuNP/carbon IDE-based biosensor, the current response of both combs were evidently increased from 0.1 V and became saturated after 0.6 V, showing the diffusion-limited current response of redox species between enzyme sites and the AuNP/carbon IDEs surface. Therefore, a 0.6 V potential was used for all subsequent experiments. In addition, for the AuNP/carbon IDE-based biosensor, the pH dependence of the biosensor was evaluated in the range of pH 5.5–8.4. As shown in Figure S9B, the maximum responses occurred at the physiological pH of 7.4 for both combs of the biosensor. Phosphate-buffered saline at pH 7.4 was thus used for measuring the current responses of AuNP/carbon IDE-based cholesterol biosensor.

### 3.5. Electrocatalytic Performance of AuNP/Carbon IDE-Based Biosensors

**Figure 5:**
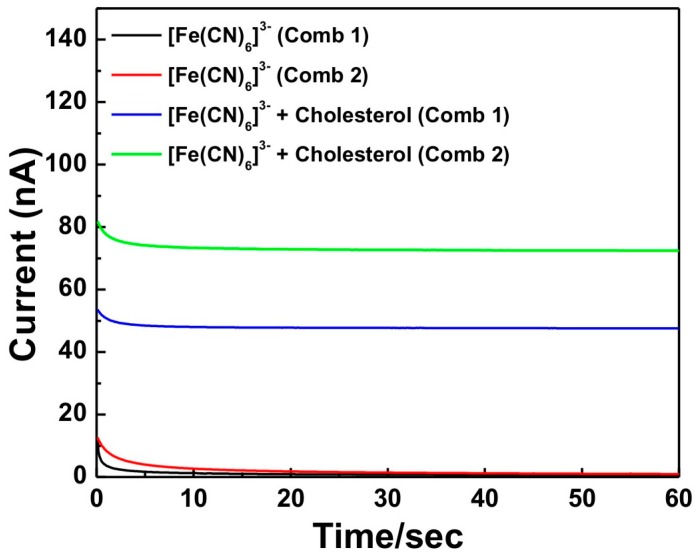
Chronoamperograms from both combs of AuNP/carbon IDEs in 0.3 mM cholesterol in 50 mM phosphate-buffered saline (PBS) containing 10 mM [Fe(CN)_6_]^3−^ (applied potential = 0.6 V vs. Ag/AgCl).

The electrocatalytic response of cholesterol at the AuNP/carbon IDE-based biosensor was measured using 10 mM [Fe(CN)_6_]^3−^ (redox mediator) under the optimized experimental conditions (applied potential = 0.6 V and pH = 7.4) via chronoamperometry (Figure 5). In the absence of cholesterol, almost no current flow was observed. However, in the presence of 0.3 mM cholesterol, the current signals reached a saturated state in less than 10 s. This signal pattern demonstrates the successful electrochemical-enzymatic redox cycling of [Fe(CN)_6_]^3*−*^/[Fe(CN)_6_]^4*−*^ between enzyme sites and the nanoelectrode (comb 1 and 2) surfaces.

**Figure 6:**
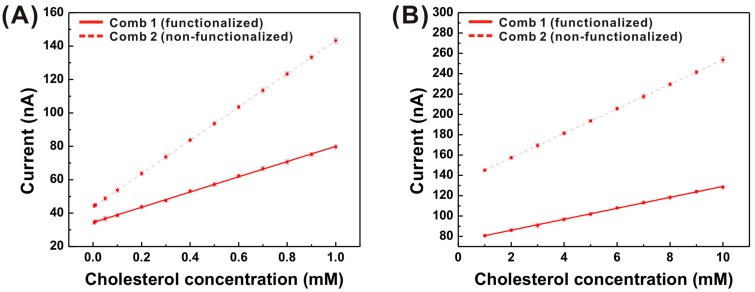
Chronoamperometric current responses of comb 1 and comb 2 of AuNP/carbon IDEs corresponding to various cholesterol concentrations: (**A**) 0.005–1 mM cholesterol, (**B**) 1–10 mM cholesterol, in 50 mM PBS containing 10 mM [Fe(CN)_6_]^3−^ (applied potential = 0.6 V vs. Ag/AgCl).

The steady-state chronoamperometric curves of cholesterol samples of various concentrations (0.005–10 mM in 10 mM [Fe(CN)_6_]^3−^/50 mM PBS (pH = 7.4) at 0.6 V vs. Ag/AgCl) were recorded for both combs (comb 1 and 2) of the AuNP/carbon IDE-based cholesterol biosensor. The current response increased with increasing cholesterol concentration, providing two linear response ranges at (i) 0.005–1 mM and (ii) 1–10 mM (Figure 6A,B, respectively). Therefore, the reported biosensor is capable of detecting cholesterol from blood and other bodily fluids such as saliva [55]. The changes in current responses and sensitivities at higher cholesterol concentration ranges were caused by the saturation of active sites of the enzymes, thereby decreasing its activity [56]. Despite the relatively low surface reactivity of comb 1 due to enzyme immobilization, comb 1 still exhibited a current response, even at low cholesterol concentrations (~5 µM). This current response is because [Fe(CN)_6_]^4−^ species are produced near the electrode surface; this enhances concentration gradient. The non-functionalized IDE comb (comb 2) exhibited a better current response, limit of detection (LOD), and sensitivity, relative to the enzyme-functionalized comb 1 of the AuNP/carbon IDE-based cholesterol biosensor (Table 1). The significantly high signal amplification at comb 2 is facilitated due to a higher electrode surface reactivity relative to comb 1.

The same experiments were executed using bare carbon IDEs selectively immobilized with ChOx, and the results were compared with those from AuNP/carbon IDEs experiments (Table 1). Compared with bare carbon IDEs, the AuNP/carbon IDEs exhibited threefold and fourfold better LOD with 1.2-fold and 1.5-fold higher cholesterol sensitivity in the concentration range of 0.005 to 1 mM and 1 to 10 mM, respectively. Additionally, a ~1.3-fold increase in current signal was also observed at AuNP/carbon IDEs for all of the cholesterol concentrations (0.005 to 10 mM). The lowest concentration detectable by the AuNP-decorated cholesterol sensor was 1.28 µM (S/N = 3). The increase in sensitivity and current signal is attributed to the reduction in linear diffusion distance between the two comb-shaped nanoelectrodes, and the increase in electrochemical activity after AuNP deposition, which facilitates the enhancement of redox cycling event of [Fe(CN)_6_]^3−^/[Fe(CN)_6_]^4−^ redox mediators.

**Figure 7:**
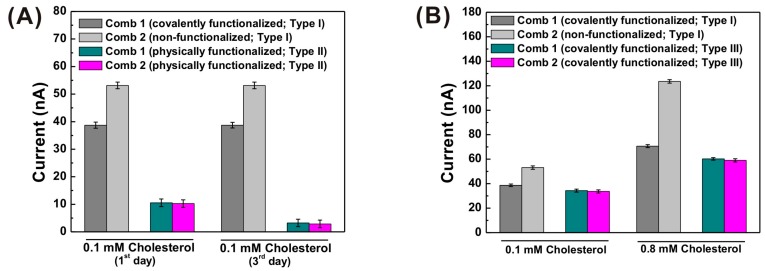
Comparison between chronoamperometric current responses of different types of AuNP/carbon IDEs (**A**) Type I and Type II, (**B**) Type I and Type III in 50 mM PBS containing 10 mM [Fe(CN)_6_]^3−^ (applied potential = 0.6 V vs. Ag/AgCl). Type I: one comb (comb 1) selectively functionalized with ChOx, Type II: all the combs physically functionalized with ChOx, Type III: all the combs covalently functionalized with ChOx.

In addition, we evaluated the effect of the spontaneously deposited ChOx on the electrode surface by measuring the chronoamperometric currents from the AuNP/carbon IDEs that were prepared without the reduction process of diazonium salt. This was so that the electrode surface condition was set to be identical to the non-functionalized comb (comb 2) of the selectively functionalized AuNP/carbon IDEs. For better understanding, the selectively functionalized IDEs using covalent binding, and the IDEs functionalized with spontaneous enzymes were denoted as Type I and Type II IDEs, respectively. As shown in Figure 7A, Type I IDEs with spontaneously deposited ChOx exhibit a much smaller current response to the cholesterol (0.1 mM) concentration compared to Type I IDEs. This confirms that the enhanced current signal at the non-functionalized comb (comb 2 of Type I IDEs) is strongly dependent on the redox cycling of the redox species between comb 2 and the enzyme sites at comb 1. Comb 1 of Type I IDEs exhibits a much higher current signal compared with both combs of Type II IDEs. This indicates that enzyme immobilization efficiency of the covalent binding process is better than spontaneous enzyme deposition. Moreover, after two days, both the physically functionalized electrodes (Type II) showed a reduction in current response, whereas the covalently functionalized electrode (comb 1) and corresponding non-functionalized electrode (comb 2) of Type I IDEs did not show any change in current response, which indicated the stability of the covalently bound enzymes.

Furthermore, as shown in Figure 7B, the AuNP/carbon IDEs at both of the combs that were covalently functionalized with ChOx (Type III IDEs) showed a smaller current response and lower sensitivity (~1.2-fold) compared with the covalently functionalized comb (comb 1) of the selectively functionalized AuNP/carbon IDEs (Type I IDEs). These results confirm the advantageous effect of the redox cycling of [Fe(CN)_6_]^3*−*^/[Fe(CN)_6_]^4*−*^ redox mediators between enzymes sites at comb 1 and the non-functionalized comb 2.

Table 2 compares the proposed AuNP/carbon IDE-based cholesterol biosensor with other reported enzyme-based cholesterol biosensors. Some of the reported cholesterol biosensors showed better sensitivity and LOD [3,16,39,58], but they exhibited limitations in the linear sensing range as, their maximum detectable concentration was below the normal physiological range (5 mM) of cholesterol. Most of the biosensors in Table 2 utilized the physical adsorption for the enzyme immobilization, whereas we have utilized the covalent binding of the ChOx enzyme, which is considered to be more stable [30,49]. The proposed AuNP/carbon IDE-based cholesterol biosensor exhibited a higher sensitivity (at both lower and higher cholesterol concentration ranges), a relatively better limit of detection, and a wider linear range as compared with other reported biosensors (Table 2). This may have been caused by the high electrical conductivity and electrochemical reactivity of AuNPs, and relatively large amounts of ChOx immobilized onto AuNP/carbon IDEs due to the large surface area, which could enhance the accessibility of the analyte to the enzyme sites in the solution, and result in an efficient redox cycling of the electron mediator.

### 3.6. Effect of AuNP Deposition on Biosensing

**Figure 8:**
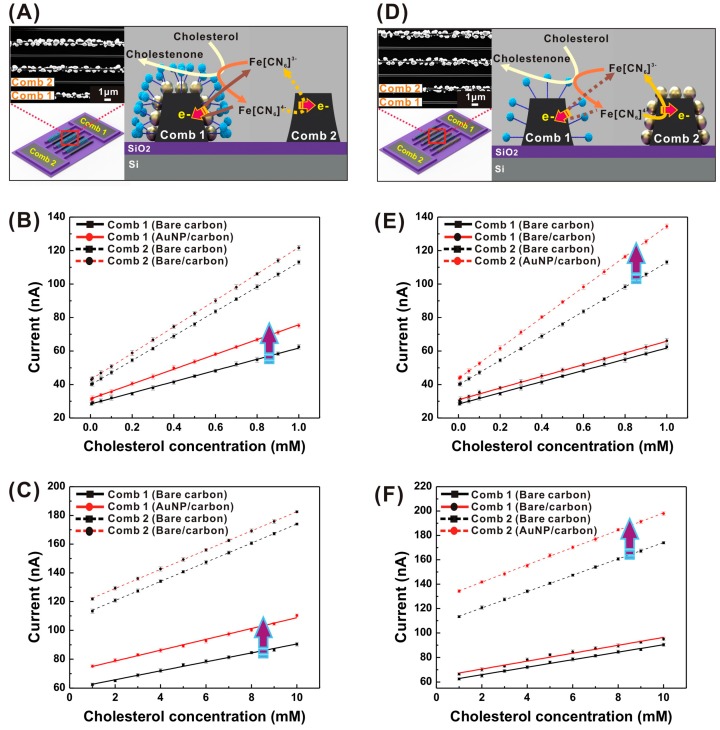
Schematic sensing mechanisms and SEM images of carbon IDEs selectively modified with AuNPs at a single comb, and those cholesterol sensing results: (**A**–**C**) comb 1: AuNP/carbon comb, comb 2: bare carbon comb; (**D**–**F**) comb 1: bare carbon comb, comb 2: AuNP/carbon comb. Calibration plots of current vs. cholesterol concentration generated in the range of (**B**,**E**) 0.005–1 mM and (**C**,**F**) 1–10 mM cholesterol in 50 mM PBS containing 10 mM [Fe(CN)_6_]^3−^ (applied potential = 0.6 V; arrows in plot indicate the enhancement in current signal, sensitivity and limit of detection (LOD) at AuNP deposited nanoelectrode).

To characterize the effect of the AuNPs on the presented biosensor performance, a single comb of carbon IDEs was selectively integrated with AuNPs, while the other comb remained as bare carbon. The cholesterol sensing experiments were performed in two different modes. First, the AuNP-integrated comb was used as comb 1 (functionalized with ChOx), and the bare carbon comb was used as comb 2 (Figure 8A). In the second mode, ChOx was immobilized in a reverse fashion (comb 1: bare carbon electrodes functionalized with ChOx; comb 2: AuNP/carbon electrodes), as shown in Figure 8D. In the first mode, when an AuNP/carbon comb was used as comb 1, both the amperometric current response and sensitivity of comb 1 increased, while comb 2 exhibited a small increase in current and almost no change in sensitivity, relative to the bare carbon IDEs (Figure 8B,C). These observations imply that the enhanced surface reactivity and electrical conductivity due to AuNP deposition play a dominant role in the enhancement of sensitivity, despite the overall increase in current signal due to the improved redox cycling between comb 1 and 2. In the second mode (Figure 8D), the amperometric current response and sensitivity of cholesterol detection were also enhanced merely at the AuNP/carbon comb (comb 2) (Figure 8E,F). This observation also confirms that fast electron transfer rates at AuNPs contribute to the enhancement of sensitivity at comb 2, while the limited surface reactivity at bare carbon electrodes (comb 1) does not significantly alter the oxidation current level. Therefore, both combs need to modify with AuNPs for the best biosensor performance, as described in Section 3.3.

### 3.7. Reproducibility of AuNP/Carbon IDE-Based Biosensor

The reproducibility of our AuNP/carbon IDE-based cholesterol biosensor was tested by measuring the current response to 0.1 mM cholesterol in 10 mM [Fe(CN)_6_]^3−^/50 mM PBS solution at a potential of 0.6 V, for five different AuNP/carbon IDE-based sensors. Both combs (combs 1 and 2) of all the five cholesterol biosensors exhibited reproducible current signals (Figure S10). The relative standard deviation of the current response for cholesterol detection at comb 1 was 1.99%, and at comb 2 was 1.58%. These observations indicate that these electrode fabrication and modification methods have appreciable reproducibility.

### 3.8. Selectivity Test and Human Serum Sample Analysis 

The selectivity of the AuNP/carbon IDE-based cholesterol biosensor was evaluated in the presence of common interfering species such as ascorbic acid (AA), uric acid (UA), salicylic acid (SA), acetaminophen (AP), glutathione (GN), glucose (GU), and creatinine (CN) (Figure S11). These interfering species were added individually with 0.1 mM cholesterol in 10 mM [Fe(CN)_6_]^3−^/50 mM PBS buffer solution, and amperometric currents were measured. The several interfering species caused slight increases in current response (AA: 7%; AP: 6.6%; UA: 3.5%; CN: 1%), whereas the addition of GN, SA, and GU did not lead to significant effects. Furthermore, when cholesterol (0.1 mM) was mixed in the solution containing all of the above interfering species (IS), we observed a ~13% increase in the current response. Thus, the AuNP/carbon IDE-based biosensor was selective for cholesterol, even at low concentration.

We tested the clinical applicability of the AuNP/carbon IDE-based cholesterol biosensor by detecting low concentrations of cholesterol in human serum. The human serum was diluted 20-fold for the determination of cholesterol in a 50 mM PBS (pH 7.4) solution. The diluted human serum solutions were afterward spiked with known concentrations of cholesterol, and measured at an applied potential of 0.6 V. The recovered ratio using this method was investigated, and the values are shown in Table S1. The AuNP/carbon IDE-based cholesterol biosensors (Table S1) exhibited good recovery, which indicates their practical feasibility as cholesterol detectors in clinical samples.

## 4. Conclusions

We have demonstrated the successful fabrication of a highly sensitive AuNP/carbon IDE-based cholesterol biosensor platform. The nano-sized carbon IDEs were fabricated by using an easy and reproducible batch process known as C-MEMS technology, and AuNPs were selectively electrodeposited on carbon IDEs using a short deposition time. Furthermore, AuNP deposition facilitated the enhancement of surface conductivity, effective surface area and efficient electron transfer, and the reduction of the inter-electrode gap that results in higher redox-cycling efficiency in AuNP/carbon IDEs compared with bare carbon IDEs. AuNP-decorated carbon IDEs could selectively functionalized with the ChOx enzyme covalently via diazonium moiety, thereby facilitating the stable configuration of an AuNP/carbon IDE-based cholesterol biosensor platform. The proposed biosensor exhibited better performances, with enhanced limits of detection of 1.28 µM, and sensitivity of 993.91 µA mM^−1^ cm^−2^, as compared with bare carbon IDEs. The good selectivity and LOD of the devices validates their application for detecting low concentrations of cholesterol from a variety of clinical samples. The highly reproducible, cost-effective and batch fabrication of nano-sized carbon IDEs, along with the high sensitivity, wide sensing range, small size, versatile modification steps, and versatile sensing mechanisms of this AuNP/carbon IDE-based biosensor platform, offer potential applicability to a variety of biosensing applications.

## Figures and Tables

**Table 1:** Comparison of sensing performance between bare carbon and AuNP/carbon IDE-based cholesterol biosensors.

Analyte	Electrode Material	Comb 1			Comb 2		
		LOD (µM)	Sensitivity (µA/(mM^.^cm^2^))	R^2^	LOD (µM)	Sensitivity (µA/(mM^.^cm^2^))	R^2^
Cholesterol (0.005–1 mM)	Bare carbon	34.31	372.55	0.989	4.15	790.75	0.998
	AuNP/carbon	22.15	468.61	0.998	1.28	993.91	0.999
Cholesterol (1–10 mM)	Bare carbon	803	31.89	0.987	110	73.48	0.997
	AuNP/carbon	234	54.80	0.998	24.6	120.29	0.999

**Table 2:** Comparison of sensing performances of the presented AuNP/carbon IDE-based cholesterol biosensor with those of various enzymatic biosensors.

Sensor Material	Linear Range (mM)	LOD (µM)	Sensitivity	Immobilization Method for ChOx	Reference
ChOx/AgNP/BDD/PAD	0.01–7	6.5	49.61 µA/(mM^.^cm^2^)	Physical adsorption	[38]
ChOx/HRP/PTH/GCE	0.025–0.125	6.3	0.18 mA/(mM^.^cm^2^)	Covalent binding	[57]
ChOx/Nafion/Bi-Pt	0.05–22	50	3.41 µA/(mM^.^cm^2^)	Physical adsorption	[5]
ChOx/CS/Ti(G) 3DNS/G	0.05–8.0	6	3.82 µA/(mM^.^cm^2^)	Physical adsorption	[14]
ChOx-ChEt/nPd-rGO/SPEs	0.005–0.14	0.05	5.12 mA/(mM^.^cm^2^)	Physical adsorption	[39]
ChOx/PANI/PVP/G	0.05–10	1	34.77 µA/(mM^.^cm^2^)	Physical adsorption	[58]
ChOx/CS/ZnO@ZnS/GCE	0.4–3	20	52.67 µA/(mM^.^cm^2^)	Physical adsorption	[11]
ChOx/PSBTz/β-CD/Graphite	0.00015–0.0225	0.005	5.77 mA/(mM^.^cm^2^)	Crosslink with CDI	[59]
ChOx-CAT/G-IL/GCE	0.00025–0.215	0.05	4.163 mA/(mM^.^cm^2^)	Physical adsorption	[16]
ChOx/Pt-Au@ZnONRs/CS-MWCNTs/GCE	0.0001–0.759	0.03	213.27 * µA/(mM^.^cm^2^)	Physical adsorption	[3]
ChOx/AuNP/carbon IDE	0.005–1	1.28	993.91 µA/(mM^.^cm^2^)	Covalent binding	This work
	1–10	24.6	120.29 µA/(mM^.^cm^2^)		

Abbreviations: ChOx, cholesterol oxidase; AgNP, silver nanoparticle; BDD, boron-doped diamond; PAD, paper-based analytical device; HRP, horseradish peroxidase; PTH, poly(thionine); GCE, glassy carbon electrode; CS, chitosan; Ti(G)3DNS/G, graphene sheets interconnected-graphene embedded titanium nanowires 3D nanostacks; G, graphene; PVP, polyvinylpyrrolidone; PANI, polyaniline; ZnO, zinc oxide; ZnS, zinc sulphide; IL, ionic liquid; nPd, dendritic Pd nanoparticle; rGO, reduced graphene; SPEs, screen printed electrode; PSBTz, poly(2-(2-octyldodecyl)-4,7-di-(selenoph-2-yl)-2*H*-benzo[*d*][1,2,3]triazole)); β-CD, β-cyclodextrin; CDI, N,N-carbonyldiimidazole; MWCNT, multi-walled carbon nanaotubes; Pt-Au, gold-platinum hybrid; ZnONRs, zinc oxide nanorods; AuNP, gold nanoparticles; IDE, interdigitated electrode. (*) Value calculated as per information in the paper.

## Supplementary Materials

The following are available online at http://www.mdpi.com/1424-8220/17/9/2128/s1.
