# Supplementary material for: Development of a Sensitive Electrochemical Enzymatic Reaction-Based Cholesterol Biosensor Using Nano-Sized Carbon Interdigitated Electrodes Decorated with Gold Nanoparticles
Source: Sensors (Basel). 2017 Sep 15;17(9):2128. doi: 10.3390/s17092128 (PMC5621016; doi:10.3390/s17092128)
Supplement: Supplementary file 1 [file sensors-17-02128-s001.pdf]

Supporting information

Development of a sensitive electrochemical-enzymatic reaction-based cholesterol biosensor using nano-sized carbon interdigitated electrodes decorated with gold nanoparticles.

D. Sharma, J. Lee, J. Seo and H. Shin*

Department of Mechanical Engineering, Ulsan National Institute of Science and Technology (UNIST), Ulsan 44919, Republic of Korea

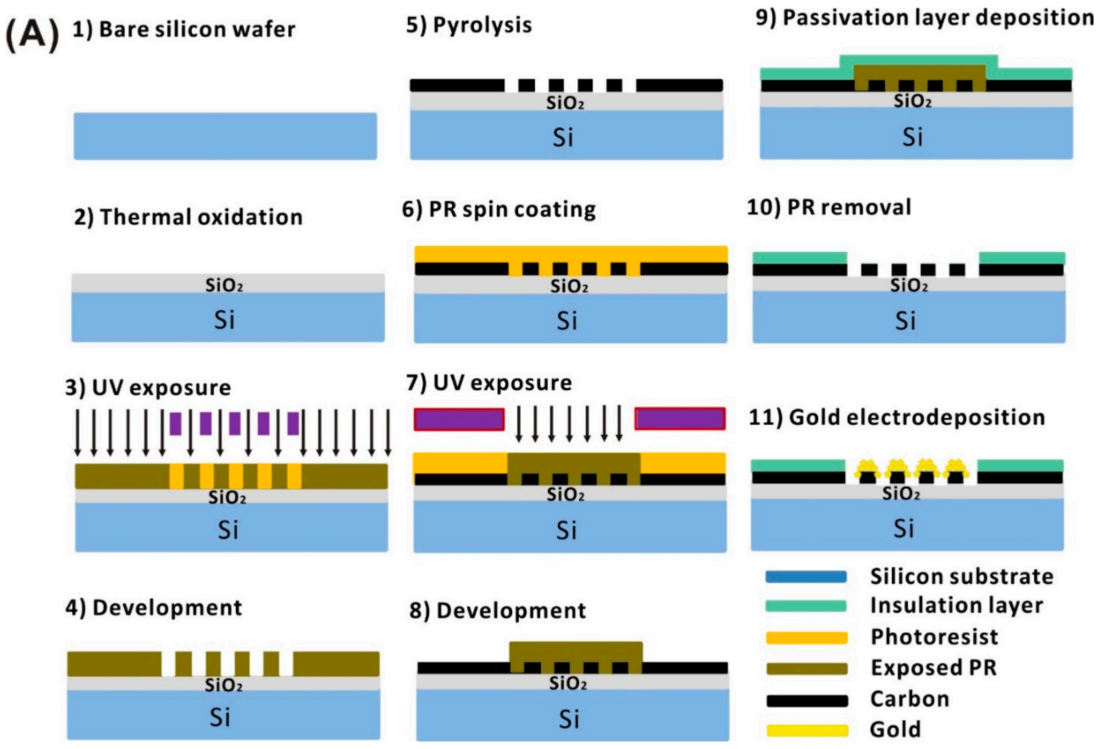

Figure S1. Schematic fabrication steps of nano-sized carbon IDE decorated with gold nanoparticles (AuNPs) using C-MEMS and electrodeposition.

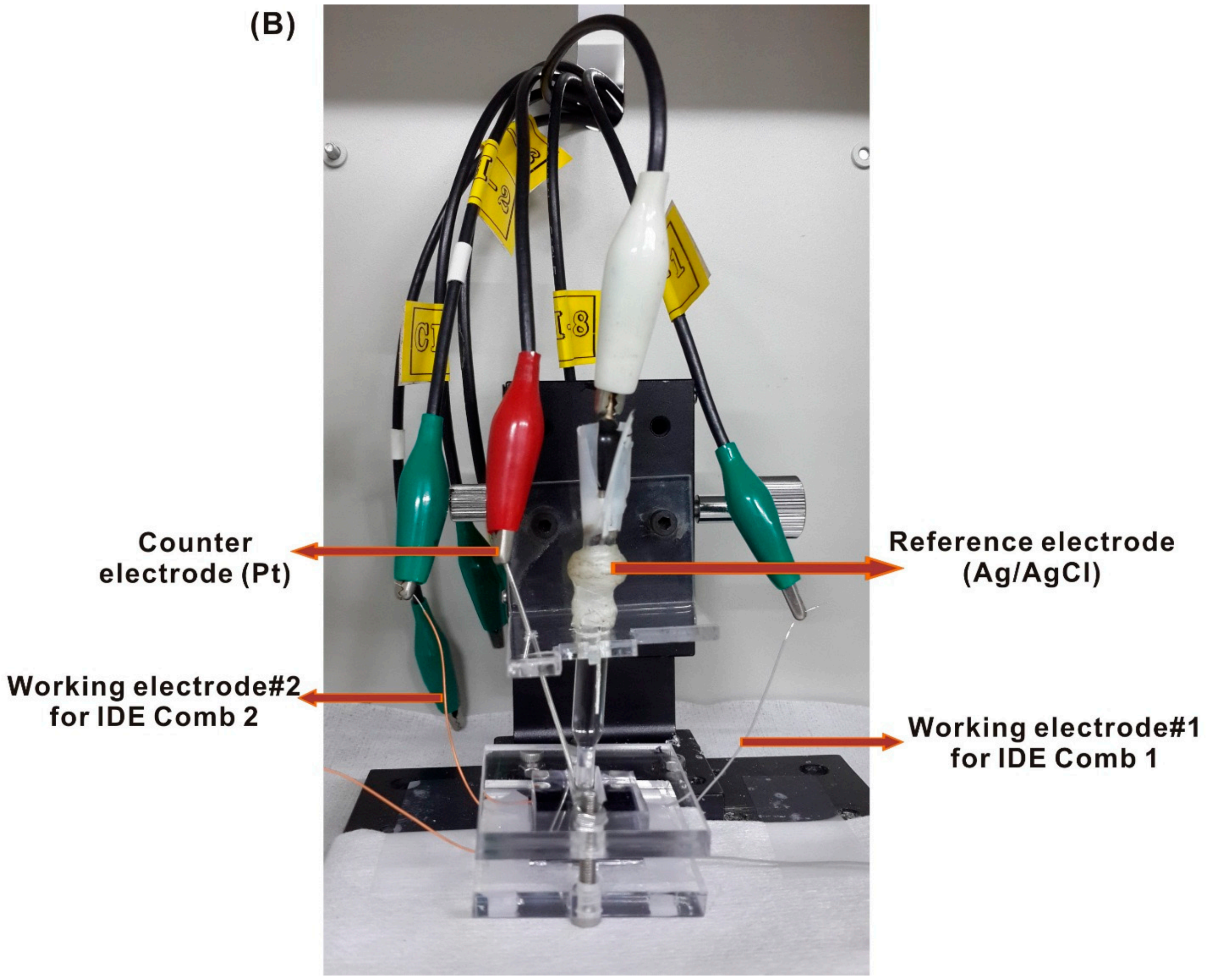

Figure S2. Setup for the electrochemical measurement using AuNP/carbon IDA nanoelectrodes.. An Ag/AgCl electrode and a Pt wire were used as the reference and counter electrodes respectively. To compare the cholesterol sensing performances between com1 and comb2, the current signals from the combs were collected separately (working electrode 1 and 2).

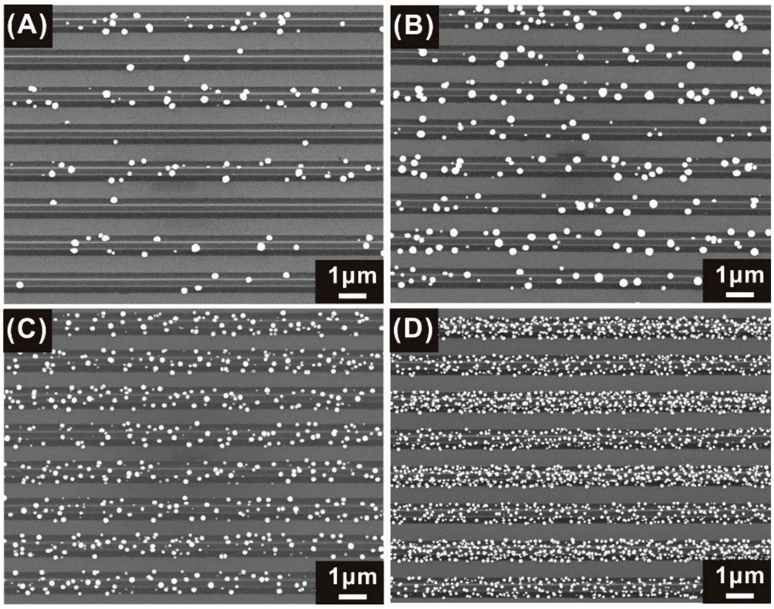

Figure S3. SEM images showing the nucleation of AuNPs onto the nano-sized carbon IDEs at various deposition conditions: (A) -0.6 V for 20 s, (B) -0.7 V for 20 s, (C) -0.8 V for 20 s, (D) -0.9 V for 20 s vs Ag/AgCl.

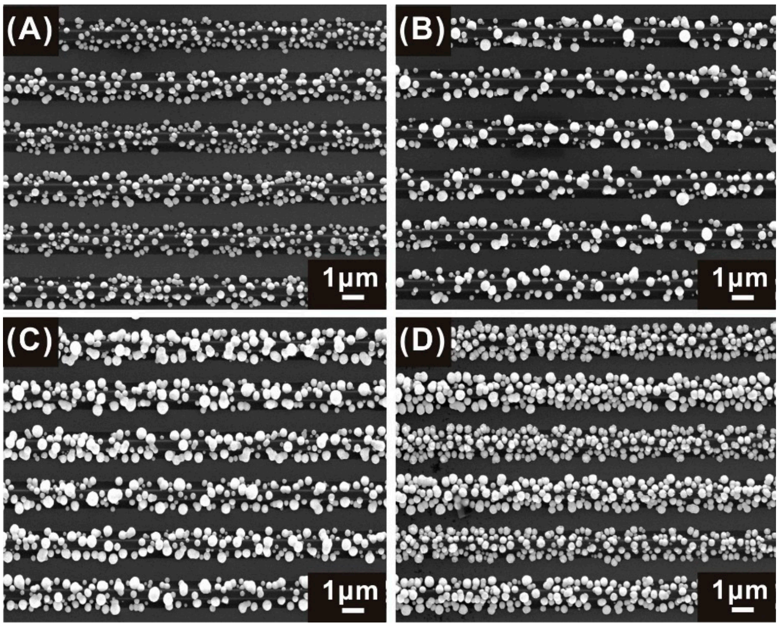

Figure S4. SEM images showing the deposition of AuNPs using -0.9 V vs Ag/AgCl as nucleation voltage for 20 s and -0.7 V vs Ag/AgCl as growth voltage for various time intervals: (A) 10 s, (B) 20 s, (C) 30 s, (D) 40 s.

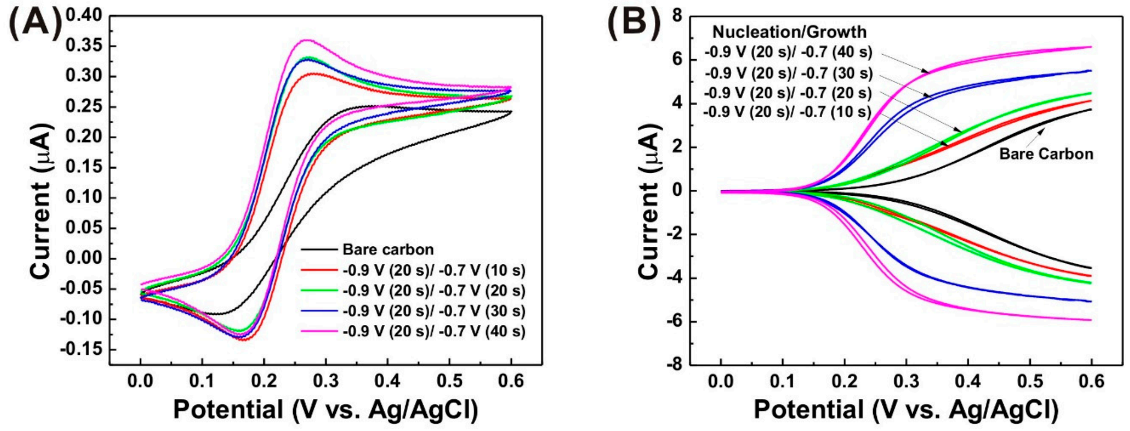

Figure S5. Cyclic voltammograms of the bare carbon IDEs and AuNP/carbon IDEs in 10 mM $[Fe(CN)_6]^{4-}$ with 0.1 M KCl. AuNPs were deposited using -0.9 V vs Ag/AgCl as nucleation voltage for 20 s and -0.7 V vs Ag/AgCl as growth voltage for various time intervals onto the nano-sized carbon IDEs: (A) single mode (without redox cycling), (B) dual mode (with redox cycling).

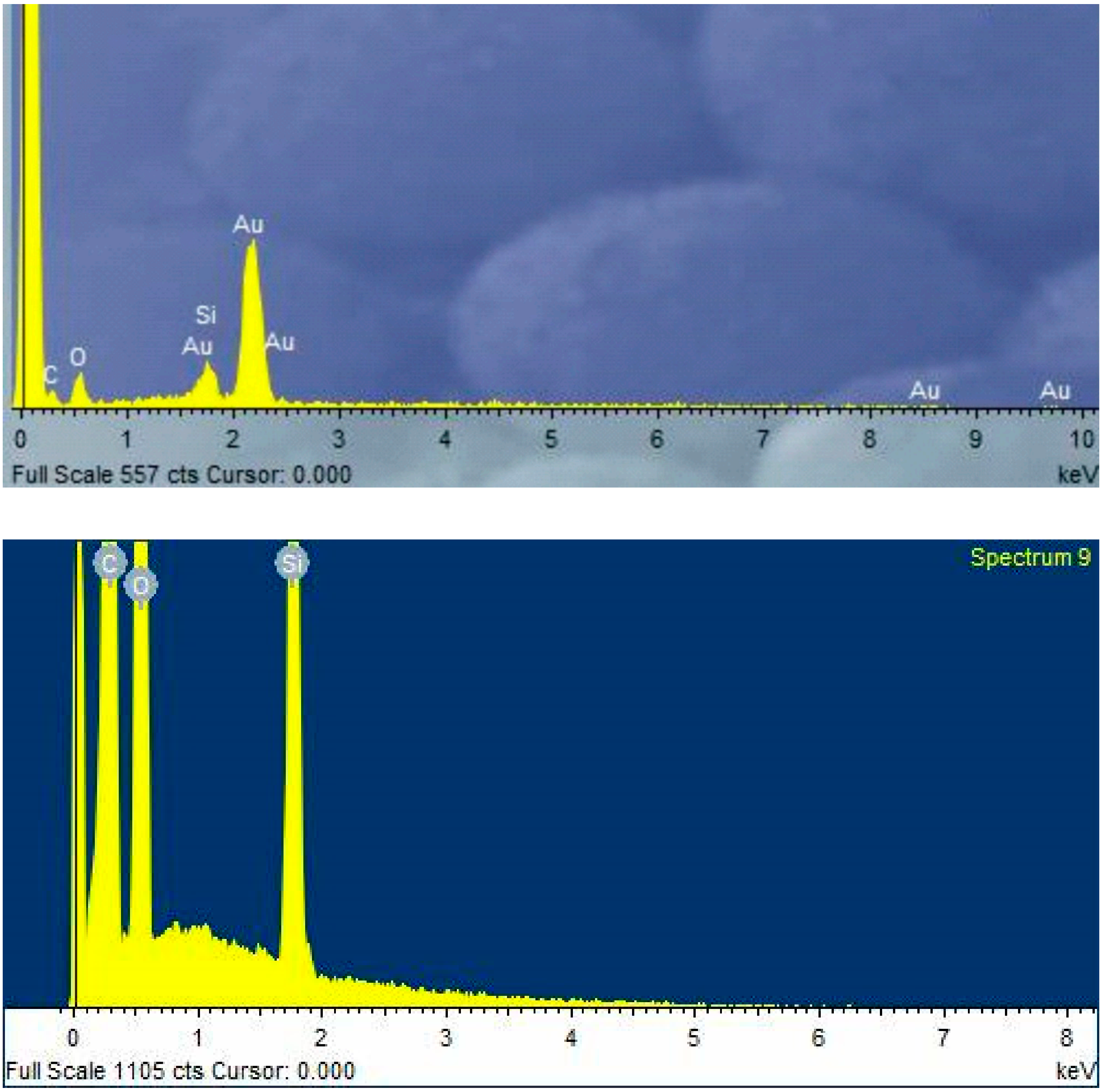

Figure S6. EDX spectrums of (top) AuNPs deposited on carbon IDEs and (bottom) bare carbon IDEs without AuNPs.

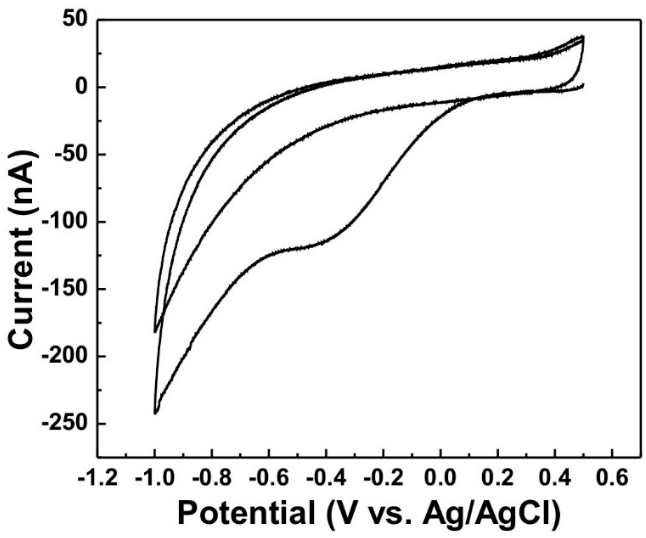

Figure S7. Cyclic voltammograms of a comb 1 of AuNP/carbon IDEs in ice cold solution containing 20 mM of 4-carboxymethylaniline (CMA), 15 mM $NaNO_2$, and 15 mM HCl at potential from 0.5 to –1.0 V vs. Ag/AgCl, and a scan rate of 200 mV/s.

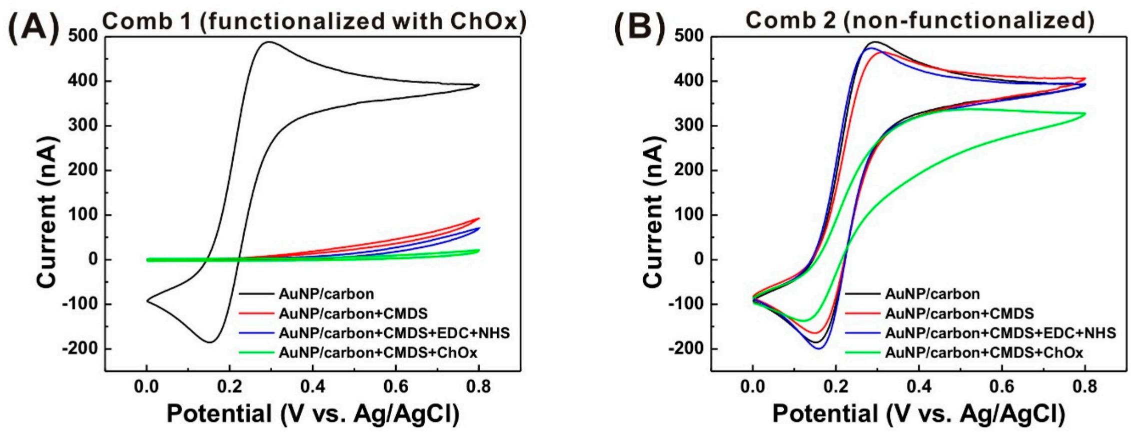

Figure S8. Cyclic voltammograms of (A) a selectively functionalized comb with ChOx (comb 1) and (B) a non-functionalized comb (comb 2) of AuNP/carbon IDEs from 10 mM $[Fe(CN)_6]^{4-}$ in 50 mM PBS after each modification step for ChOx immobilization on comb 1; CMDS: 4-Carboxymethyl diazonium salt, EDC: 1-Ethyl-3-(3-dimethylaminopropyl)-carbodiimide, NHS: N-Hydroxysuccinimide, ChOx: Cholesterol oxidase.

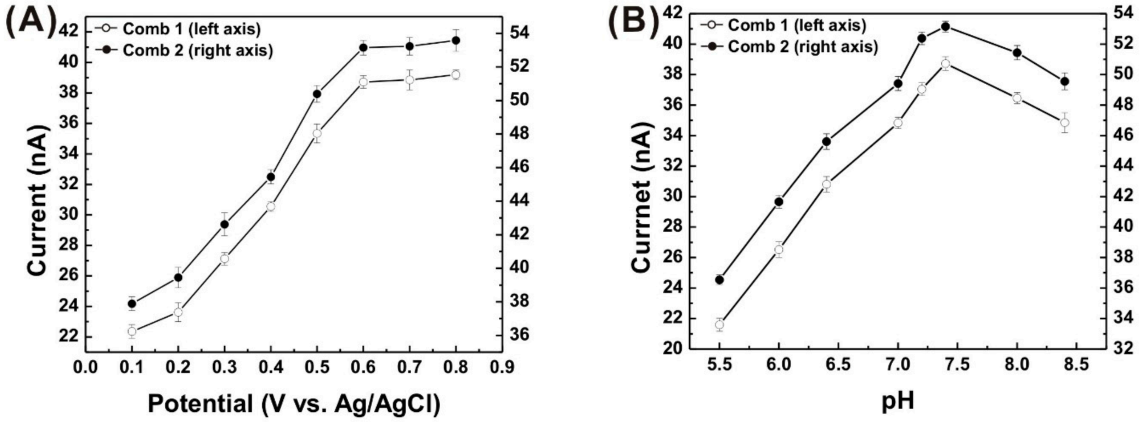

Figure S9. Effects of (A) applied potential (pH = 7.4) and (B) PBS buffer pH (applied potential = 0.6 V vs. Ag/AgCl) on amperometric current signals for 0.1 mM cholesterol from combs 1 and 2 of a AuNP/carbon IDE-based biosensor using 10 mM $[Fe(CN)_6]^{3-}$/50 mM PBS.

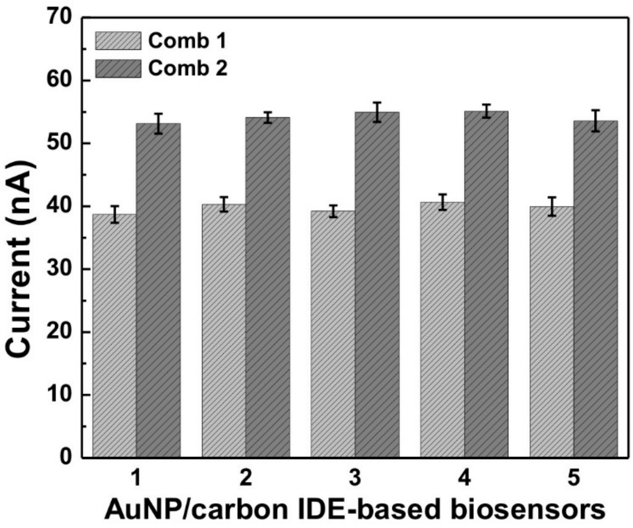

Figure S10. Amperometric current responses at combs 1 and 2 of five different AuNP/carbon IDE-based biosensors for 0.1 mM cholesterol, in 50 mM PBS containing 10 mM $[Fe(CN)_6]^{3-}$ solution.

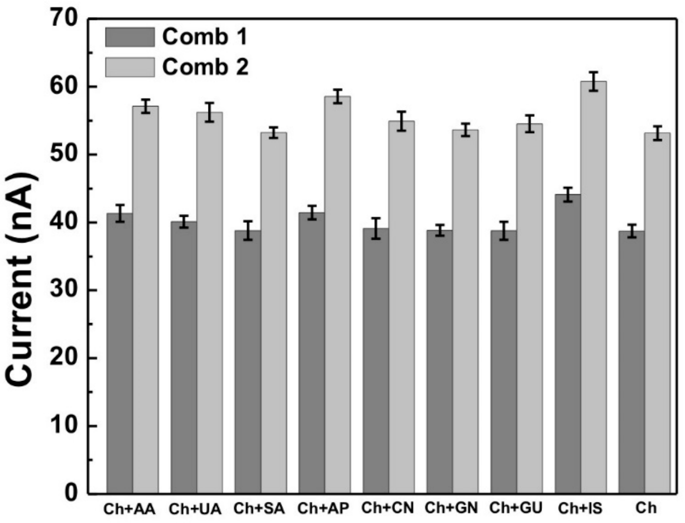

Figure S11. Amperometric current responses of AuNP/carbon IDEs in the presence of interfering species (AA: 1 mM ascorbic acid; UA: 1 mM uric acid; SA: 1.5 mM salicylic acid; AP: 1 mM acetaminophen; CN: 1.5 mM creatinine; GN: 1 mM glutathione; GU: 5 mM glucose; IS: interfering solution containing all species), measured along with 0.1 mM cholesterol (Ch) in 50 mM PBS containing 10 mM $[Fe(CN)_6]^{3-}$.

Table S1. Cholesterol estimation in human blood serum using a AuNP/carbon IDE-based biosensor.

Analyte	Sample number	Concentration in serum solution (mM)	Concentration detected (mM)[b]	Recovery (%)[c]
**Cholesterol**	1	0.37	0.40	108.1
	2	0.57 [a]	0.54	94.7
	3	0.78 [a]	0.82	105.1
	4	1.18 [a]	1.26	106.7
	5	2.19 [a]	2.22	101.3

[a] Human serum solution spiked with standard solution

[b] Average of three determinations

[c] Recovery = (Concentration determined by sensor) / (Concentration in serum) × 100
